# NG2‐Glia in Central Nervous System Following Injury: From Pathological Involvement to Repair Potential

**DOI:** 10.1002/cns.71162

**Published:** 2026-09-15

**Authors:** Yunyun Cai, Xin Shen, Ming Li, Junjie Li, Gang Chen

**Affiliations:** ^1^ Department of Histology and Embryology Medical School of Nantong University, Co‐Innovation Center of Neuroregeneration Nantong Jiangsu China; ^2^ Department of Anesthesiology Affiliated Hospital of Nantong University Nantong Jiangsu China

**Keywords:** CNS injury, glial cells, immune regulatory, neuron, NG2‐glia, repair

## Abstract

**Background:**

Central nervous system (CNS) injury remains a severe neurological condition with limited regenerative capacity. NG2‐glia are abundant and functionally versatile glial cells that have attracted increasing attention in CNS repair research. However, their roles are complex and context‐dependent, ranging from early pathological involvement to later reparative potential, and a systematic synthesis of this temporal dichotomy is lacking.

**Method:**

We systematically searched PubMed and Web of Science from inception to 2026 using combinations of keywords including “NG2‐glia,” “NG2 cells,” “oligodendrocyte precursor cells,” “OPCs,” and “CNS injury.” Additional references were identified by manual screening of reference lists. We prioritized mechanistic and therapeutic studies published within the last 10 years, while foundational discoveries were included for historical context.

**Results:**

This review establishes that NG2‐glia exhibit a time‐dependent dual role after CNS injury. In the early stage (hours to days), they rapidly proliferate, migrate to the lesion, and secrete chondroitin sulfate proteoglycans (CSPGs), forming a chemical and physical barrier that inhibits axonal regeneration and may facilitate neuropathic pain. In the middle‐to‐late stages (days to weeks/months), they shift toward repair functions, including remyelination via differentiation into oligodendrocytes, neuronal trans‐differentiation (primarily under forced reprogramming), and anti‐inflammatory/neuroprotective effects through interactions with microglia, astrocytes, and neurons. Their fate is determined by intrinsic factors (age, heterogeneity) and extrinsic microenvironmental signals (damage‐associated molecules, cytokines, and signaling pathways such as Wnt/β‐catenin and TGF‐β2). Therapeutically, diverse strategies—including CB1 receptor agonists to promote remyelination, chondroitinase ABC to neutralize CSPG inhibition, and SOX2/NeuroD1‐based reprogramming to generate functional neurons—have shown preclinical promise, yet significant translational gaps remain, including poor endogenous neurogenesis, constitutive differentiation limitations, and safety concerns.

**Conclusion and Perspective:**

Targeting NG2‐glia offers a novel avenue for CNS repair, but successful interventions must be precisely timed and stage‐specific rather than unregulated promotion or inhibition. This review proposes an analytical framework of “time‐dependent dual roles” to reconcile the contradictory functions of NG2‐glia and highlights critical future directions, including deciphering regional heterogeneity, elucidating the molecular switches that govern state transitions, validating the functional integration of reprogrammed neurons, and bridging the bench‐to‐bedside divide. A deeper understanding of the regulatory logic underlying NG2‐glia plasticity will inform next‐generation precision therapies for CNS injuries.

## Graphical abstract

Following CNS injury, NG2‐glia display distinct functional phases. In the early stage, NG2‐glia are rapidly activated, proliferate, migrate to the lesion site and secrete CSPGs, which form a chemical and physical barrier that inhibits axonal regeneration. In the middle to late stage, NG2‐glia transition toward repair functions, such as remyelination, neuronal trans‐differentiation, and anti‐stress/protective effects. This figure was created with BioRender.com (https://www.biorender.com).

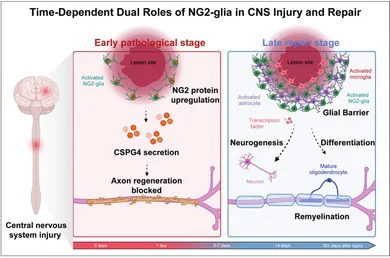

**Abbreviations**
ALS: amyotrophic lateral sclerosis
Ascl1: achaete‐scute family bHLH transcription factor 1
ATP: adenosine triphosphate
BBB: blood–brain barrier
Bcl‐2: B‐cell lymphoma 2
CD11b: cluster of differentiation 11b
CMR: contact‐mediated repulsion
CNS: central nervous system
CSPG: chondroitin sulfate proteoglycan
DAMPs: diverse damage‐associated molecular patterns
DSCAMs: down syndrome cell adhesion molecules (DSCAMs)
EAE: experimental autoimmune encephalomyelitis
GABA: gamma‐aminobutyric acid
GFAP: glial fibrillary acidic protein
GPR17: G protein‐coupled receptor 17
HGF: hepatocyte growth factor
HMGB1: high mobility group box 1
IL‐1β: interleukin‐1β
LAR: leukocyte common antigen‐related receptor
LINGO1: leucine‐rich repeat and Ig domain‐containing Nogo receptor‐interacting protein 1
LPS: lipopolysaccharide
MAP 2: microtubule‐associated protein 2
MCAO: middle cerebral artery occlusion
MMPs: matrix metalloproteinases
MYC: MYC proto‐oncogene, bHLH transcription factor
NeuN: neuronal nuclei
NG2: neuron‐glial antigen 2
Nrp1: neurociliin‐1
OASIS: old astrocyte specifically induced substance
OPCs: oligodendrocyte precursor cells
PDGFRα: alpha‐type platelet‐derived growth factor receptor
PNI: peripheral nerve injury
PTPσ: protein tyrosine phosphatase sigma
SCI: spinal cord injury
SOX2: SRY‐box transcription factor 2
TBI: traumatic brain injury
TGF‐β2: transforming growth factor‐β2
Th2: T‐helper Type 2
TIMP‐1: tissue inhibitor of metalloproteinase‐1
TLR4: toll‐like receptor 4
TNF‐α: tumor necrosis factor α

## 1 Introduction

Central nervous system (CNS) injury represents a severe neurological condition, primarily due to the limited regenerative capacity of neurons and myelinating oligodendrocytes [1, 2]. Consequently, existing therapies for central neuropathy often achieve only minimal efficacy in promoting functional neural regeneration [3, 4, 5]. Recent evidence indicates that CNS repair and regeneration involve not only neurons but also glial cells—including microglia, astrocytes, and oligodendrocytes—that actively participate in neural network remodeling following injury [6, 7, 8]. As the most abundant cell population in the CNS, glial cells contribute to post‐injury repair processes [9], immune response [10], and substance metabolism [11] following injury.

NG2‐glia, also widely referred to as oligodendrocyte precursor cells (OPCs) or NG2 cells, represent a major glial population in the CNS and are the focus of this review. We note that chondroitin sulfate proteoglycan 4 (CSPG4)/neuron‐glial antigen 2 (NG2) expression is not entirely restricted to OPC‐lineage cells under certain injury conditions (e.g., pericytes and some macrophage subsets can transiently express NG2), and this caveat is considered throughout the review when interpreting NG2^+^ cell identity. Initially identified solely as progenitors of oligodendrocytes, NG2‐glia are now recognized to possess additional functions, including the capacity to generate astrocytes and neurons under certain conditions [12, 13], as well as serving as the primary source of remyelinating cells in demyelinated lesions [14]. Following CNS injury, NG2‐glia rapidly proliferate and migrate toward the injury site [15, 16, 17]. These reactive NG2‐glia exhibit altered expression of numerous signaling molecules, thereby modulating neuroimmune responses and occupying a critical position in the recovery process [18, 19]. However, their dense clustering can also impede axonal elongation within glial scars after spinal cord injury (SCI) [20, 21]. Owing to these multifaceted roles, NG2‐glia have gained increasing research attention over the past decade, with about 300 publications addressing this topic. To ensure a comprehensive and up‐to‐date coverage of the literature, we searched PubMed and Web of Science from inception to 2026 using combinations of the following keywords: “NG2‐glia,” “NG2 cells,” “oligodendrocyte precursor cells,” “OPCs,” and “CNS injury.” Additional relevant references were identified by manually screening the reference lists of retrieved articles and key reviews. For mechanistic and therapeutic discussions, we prioritized studies published within the last 10 years (2016–2026), while foundational discoveries (e.g., the identification of NG2‐glia by Raff et al., 1983) [22] are included as essential historical context. The final reference list was curated to balance comprehensiveness and relevance, with particular emphasis on studies that inform the time‐dependent dual‐role framework proposed in this review. On the basis of this growing body of evidence, NG2‐glia are now recognized as key players in various CNS injuries, including ischemic injury [23], cerebral small vessel disease [24] and acute brain trauma [25] and SCI [12].

The understanding of NG2‐glia is evolving from perceiving them as “single‐lineage precursor cells” to recognizing them as “multifunctional damage‐responsive cells.” The time‐dependent dual‐role framework proposed in this review explicitly distinguishes the early‐stage pathological involvement of NG2‐glia (e.g., CSPG secretion, neuropathic pain facilitation) from their middle‐to‐late‐stage repair potential (e.g., remyelination, neurogenesis, neuroprotection). This time‐dependent functional dichotomy raises a fundamental question: what determines the transition of NG2‐glia from a pathological to a reparative phenotype? Age, microenvironmental signals, and key transcription factors jointly determine the fate of NG2‐glia through a complex regulatory network. The above mechanistic understanding has direct implications for therapeutic design. Given the time‐dependent nature of NG2‐glia functions, interventions should not simply promote or inhibit NG2‐glia activity in an unregulated manner. Instead, therapeutic strategies should precisely regulate the timing and mode of intervention according to the post‐injury stage.

## 2 What are NG2‐Glia?

### 2.1 Discovery, Distribution and Phenotypic Characteristics of NG2‐Glia

**FIGURE 1:**
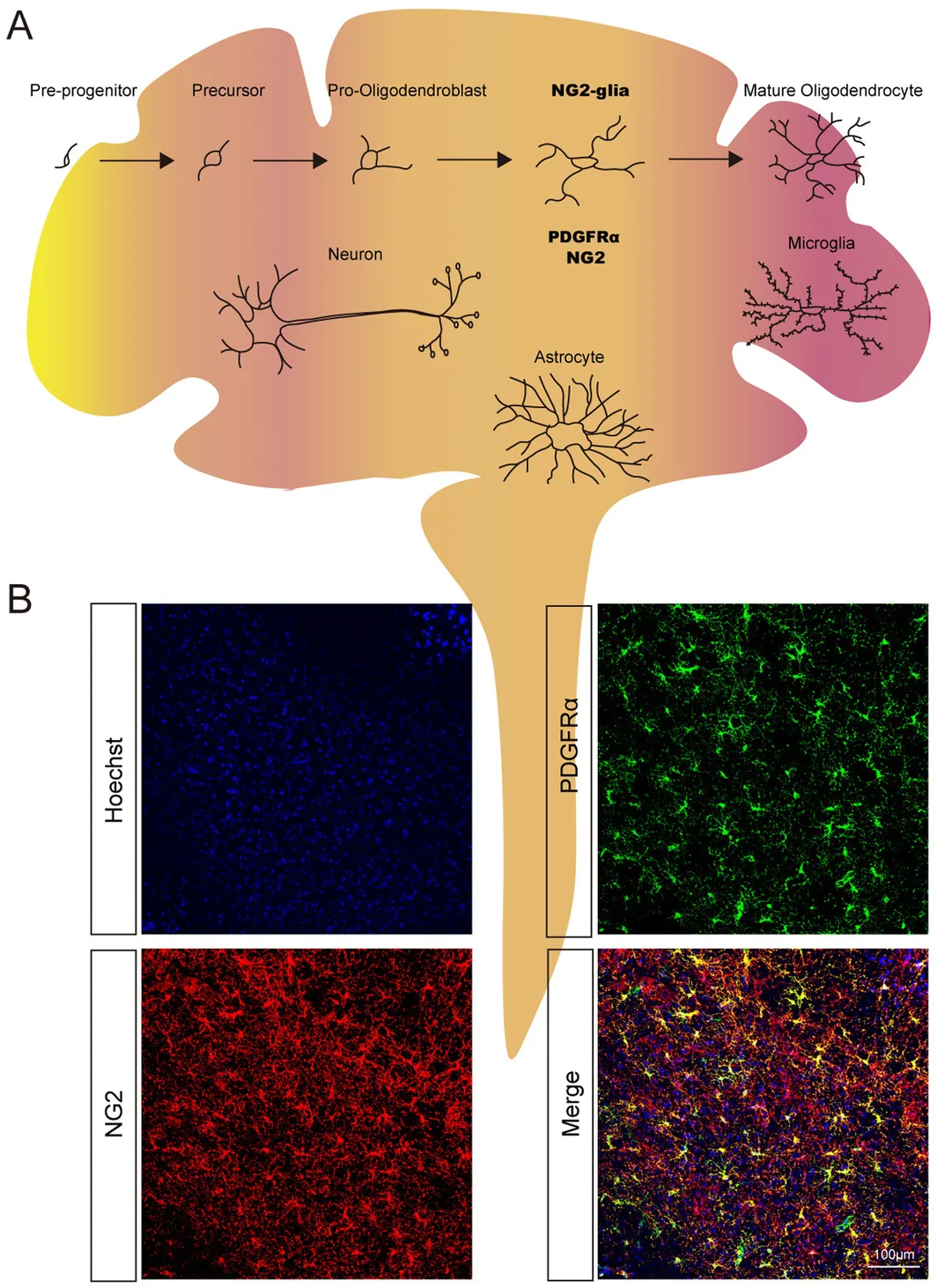
Lineage and identification of NG2‐glia. (A) Schematic diagram of the cellular composition of the CNS, depicting the progression of the oligodendrocyte lineage from pre‐progenitor cells to mature oligodendrocytes. (B) Representative immunofluorescence images of NG2‐glia (NG2^+^/PDGFRα^+^) in the adult mouse spinal cord. To accurately label NG2‐glia, we typically employ the approach of co‐staining NG2 (red, rabbit, 1:200; Millipore, catalog: AB5320) with PDGFRα (green, goat, 1:50; R&D Systems, catalog: AF1062—SP). Hoechst (blue, Beyotime, P0133) is used to label nuclei. The scale bar represents 100 μm. Among these markers, PDGFRα is a canonical marker used to identify NG2‐glia and mediates PDGF‐AA‐dependent proliferation and migration signaling essential for their maintenance and injury response. The immunofluorescence images shown in the figure are original data generated by the authors' laboratory.

NG2‐glia, also known as O2A type oligodendrocyte‐astrocyte progenitors, were initially discovered in 1983 by Raff et al. in the optic nerve as a distinct cell population capable of differentiating into either oligodendrocytes or astrocytes under specific in vitro culture conditions [22]. In the CNS, NG2‐glia are uniformly distributed in the gray matter and the white matter, forming non‐overlapping “exclusive domains” [26]. Although some debate remains regarding their classification as a fully distinct lineage, it is now widely accepted that NG2‐glia constitute a widely distributed and functionally versatile glial population in the CNS, alongside astrocytes, microglia, and oligodendrocytes [24] (Figure 1A). NG2‐glia are commonly identified by co‐staining with two markers, NG2 and alpha‐type platelet‐derived growth factor receptor (PDGFRα), as depicted in the following figure (Figure 1B).

### 2.2 Physiological Functions of NG2‐Glia

Proliferation stands out as the most prominent characteristic of adult NG2‐glia. Nearly all NG2‐glia possess proliferative potential [27, 28]. NG2‐glia migration commences in the early embryonic stage and depends on the vascular system as a physical support structure [27, 29]. Contact‐mediated repulsion (CMR) is mediated by bidirectional signaling transmembrane proteins (such as Eph‐ephrin, down syndrome cell adhesion molecules (DSCAMs), leucine‐rich repeat and Ig domain‐containing Nogo receptor‐interacting protein 1 (LINGO1), etc.) to guarantee that NG2‐glia maintain a non‐overlapping spatial distribution, thus maintaining cellular homeostasis [30, 31, 32]. However, the newly generated NG2‐glia need to maintain a stable total cell number by differentiating into oligodendrocytes or undergoing apoptosis. This capacity is essential for axon myelination, providing metabolic and functional support to neurons [33]. Moreover, NG2‐glia express a variety of voltage‐ and ligand‐gated channels on their membranes and form synapses with neurons [34, 35, 36, 37]. This interaction between neurons and glial cells suggests that NG2‐glia may play a role in the functional regulation of neural circuits. Although NG2‐glia continuously generates a small amount of new oligodendrocytes in adults to maintain myelin remodeling and repair, the overall oligodendrocytes population remains highly stable (only approximately 0.3% of oligodendrocytes in humans are replaced each year, and around 90% of oligodendrocytes in mice can survive for more than 8 months) [38, 39, 40]. Based on this, it can be inferred that under physiological conditions, apart from differentiating into oligodendrocytes, NG2‐glia may undertake other important yet undefined functions, especially when the nervous system is damaged or diseased.

## 3 Regulatory Network Governing NG2‐Glia Fate Determination

### 3.1 Internal Determinants: Age and Heterogeneity

Age is a crucial factor that determines the fate of NG2‐glia [41]. The transition from a highly proliferative state during the developmental stage to a static and even senescent state in adulthood fundamentally constrains their regenerative potential [42]. A recent transcriptomic study comparing fetal and adult NG2‐glia revealed aging is not a passive functional decline but an active inhibitory process driven by a transcription factor network centered on MYC proto‐oncogene, bHLH transcription factor (MYC) [43]. In addition, age‐related microenvironmental changes also have a profound impact on the fate of NG2‐glia. Another study on the human brain reveals that during normal aging, the level of NG2 protein and hepatocyte growth factor (HGF), which promotes nerve regeneration, increase in a linear manner [44]. This might be a compensatory protective mechanism of the body. However, under pathological conditions (such as depression), this balance is disrupted, characterized by elevated neuroinflammatory markers (interleukin‐1β [IL‐1β], tumor necrosis factor α [TNF‐α]), and the relationship between NG2 and the anti‐apoptotic factor B‐cell lymphoma 2 (Bcl‐2) is decoupled. In addition, the susceptibility of white matter NG2‐glia to ischemic injury varies with age: young mice exhibit greater resistance to ischemic damage in NG2‐glia, whereas adult mice have a significantly reduced number of labeled viable NG2‐glia 24 h after middle cerebral artery occlusion (MCAO) modeling [45].

NG2‐glia exhibit significant heterogeneity under both physiological and injury conditions [46, 47]. Wang et al. [26] introduced the “reactive OPC state code” framework, proposing that reactive OPCs are dynamic regulatory units—not merely passively activated cells—that adopt distinct transcriptional, metabolic, and functional states in response to injury signals. Current research shows that reactive NG2‐glia behavior depends on injury type: after traumatic brain injury (TBI) or spinal cord injury, they exhibit the strongest proliferative response—accounting for ~60% of all proliferating cells within 24 h post‐injury [27, 48]. Some NG2‐glia even express the microglia/macrophage markers cluster of differentiation 11b (CD11b) and CD68, which implies that they possess immune‐like functions [38, 49, 50]. In ischemic injury, NG2‐glia responses vary by region and time: their numbers drop sharply in the infarct core, while cells in the peri‐infarct zone undergo hypertrophy and develop thicker processes [51]. Under neuroinflammatory conditions, lipopolysaccharide (LPS) can indirectly activate NG2‐glia via Toll‐like receptor 4 (TLR4), yet excessive inflammation can also impede the differentiation and migration of NG2‐glia [52, 53]. Reactive NG2‐glia show a distance‐dependent injury response: those near the injury site adopt a bipolar, elongated migratory morphology; those farther away display an enlarged soma, upregulated NG2 expression, and increased dendritic branching [27, 48, 54]. In summary, the heterogeneity of NG2‐glia is reflected in multiple dimensions of their response states, including fate bias, functional status, driver signals, injury types, and injury distances.

### 3.2 External Micro‐Environment Signals

The reactive state of NG2‐glia is precisely regulated by complex microenvironmental signals after CNS injury [19, 55]. After neural injury, necrotic neurons in the lesion core release diverse damage‐associated molecular patterns (DAMPs)—including IL‐1α, IL‐33, adenosine triphosphate (ATP), high mobility group box 1 (HMGB1), and glutamate—that directly or indirectly activate NG2‐glia [48, 56, 57]. Blood–brain barrier (BBB) disruption facilitates substantial infiltration of plasma‐derived proteins—such as fibrin, fibrinogen, and collagen—which induce reactive phenotypic changes in glial cells, particularly NG2‐glia [57]. Upon in vitro stimulation, NG2‐glia upregulate various proinflammatory chemokines, cytokines, and matrix metalloproteinases (MMPs) [58]. At the molecular level, injury activates the Wnt/β‐catenin signaling pathway, which stimulates the proliferation of NG2‐glia and contributes to glial scar formation [59]. GPR17 is upregulated in reactive NG2‐glia, facilitating their recruitment to lesion sites and promoting their differentiation into myelinating oligodendrocytes—demonstrated consistently in both needle‐puncture and cerebral ischemia models [29, 60, 61, 62]. Furthermore, post‐ischemic dysregulation of potassium (K^+^) channel homeostasis promotes NG2‐glia proliferation [27, 63, 64], whereas transforming growth factor‐β2 (TGF‐β2)/TGF‐β Type II receptor (TGFBR2) axis modulates NG2‐glia‐mediated maintenance of blood–brain barrier integrity [18].

### 3.3 Other Cell Types Interacting With NG2‐Glia in CNS Repair

**FIGURE 2:**
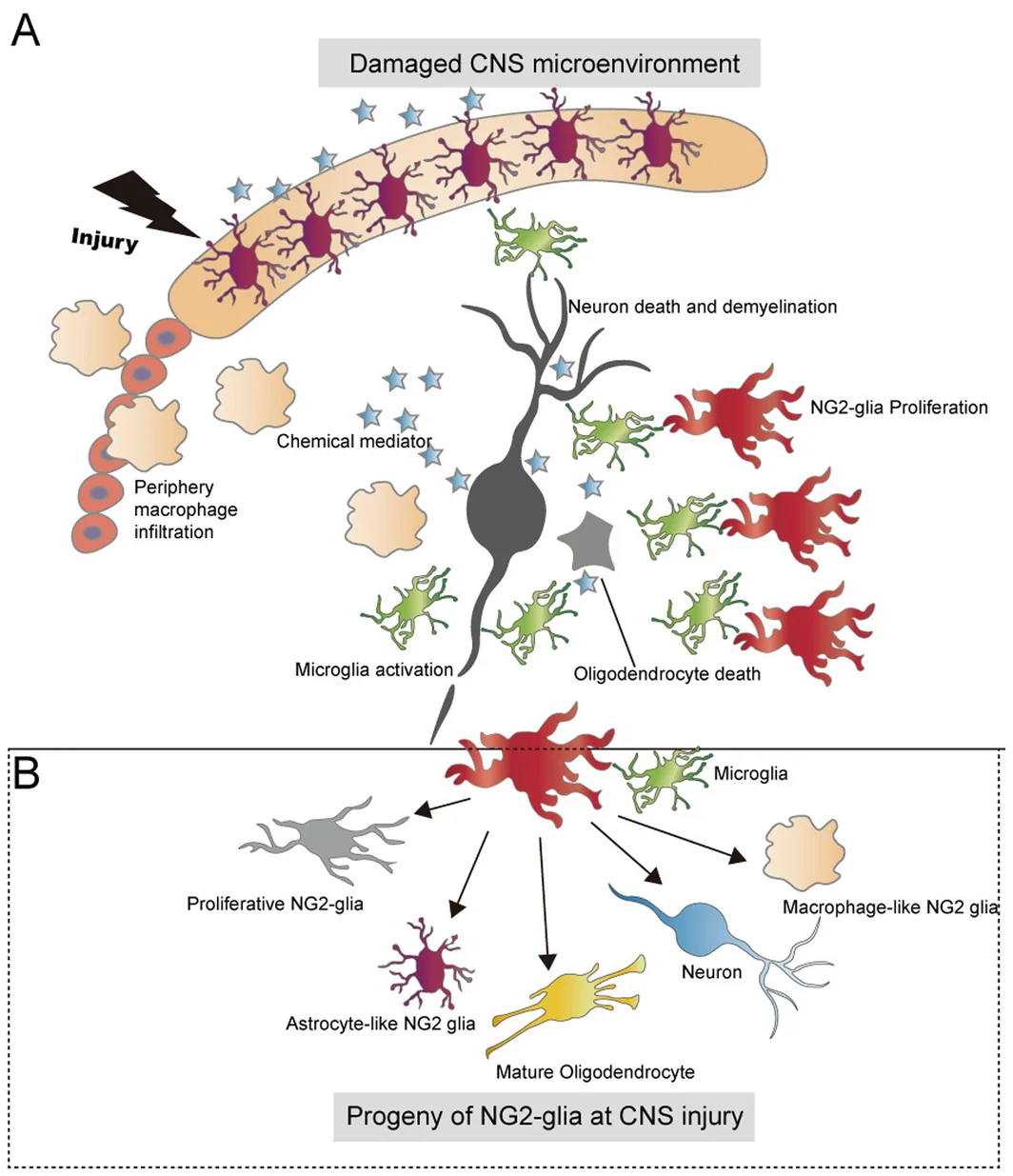
Intercellular interaction network of NG2‐glia following CNS injury. (A) Schematic of the damaged CNS microenvironment, depicting the spatial relationships among neurons, astrocytes, microglia, oligodendrocytes, NG2‐glia, and infiltrating macrophages in the lesion core and peri‐lesion areas. The lesion core is characterized by cell loss and an inflammatory milieu, while the peri‐lesion zone harbors reactive NG2‐glia and activated glial cells. (B) NG2‐glia exhibit multilineage differentiation potential (oligodendrocytes, astrocytes, and neurons) and engage in bidirectional crosstalk with microglia/macrophages, modulating immune responses and tissue repair. This figure was created with Adobe Illustrator 2025.

NG2‐glia not only participate in CNS injury responses but also actively shape repair through extensive interactions with other cells (Figure 2).

#### 3.3.1 Astrocytes

A recent study revealed a mechanism linking astrocyte foot process formation to NG2‐glia migration and differentiation under physiological conditions: after NG2‐glia reach their destination, astrocyte‐derived foot processes on blood vessels trigger their detachment, enabling differentiation into mature oligodendrocytes [65].

Previous studies show that the glial scar consists of diverse activated astrocytes [66, 67], and NG2‐glia may contribute to cellular diversity in the injured CNS by differentiating into astrocytes [16]. However, whether NG2‐glia are a significant source of reactive astrocytes is a matter of longstanding debate that is highly dependent on injury type and microenvironment. Under physiological conditions or in certain injury models (such as stab wounds), lineage‐tracing studies have shown that the proportion of NG2‐glia differentiating into astrocytes is extremely low, suggesting they are not a major source of reactive astrocytes in the adult CNS [68, 69]. Nevertheless, this plasticity can be induced in specific injury environments. For example, approximately 25%–40% of NG2‐glia exhibit signs of astrocytic differentiation one month after SCI, and these derived cells are heterogeneous; in contrast, the proportion of NG2‐glia undergoing astrocyte differentiation within experimental autoimmune encephalomyelitis (EAE) lesions is estimated to be around 9% [46]. Furthermore, inflammatory factors (as seen in ALS models) can prompt NG2‐glia to differentiate into astrocytes [70], while knockdown of β‐catenin reduces NG2‐glia‐derived astrocytes, thereby attenuating glial scar formation and promoting axonal regeneration [71]. These findings suggest that NG2‐glia‐to‐astrocyte differentiation is not an inherent default fate but rather an injury‐type‐dependent plasticity, the extent and functional significance of which vary across pathological contexts. Notably, although astrocytes are largely perceived as inhibiting recovery after SCI [72], they may actually serve multiple advantageous purposes [73]; astrocytes can also contribute to myelin formation after EAE [74]. Furthermore, recent investigations have demonstrated that astrocyte‐like NG2‐glia can temporarily take over astrocytic functions near lesions, since few astrocytes divide, proliferate, or migrate in that area [75]. Proliferating NG2‐glia represents the initial crucial participant in tissue remodeling and wound healing following acute brain injury [76].

#### 3.3.2 Microglia/Macrophages

After CNS injury, there is a complex and dynamic bidirectional relationship between NG2‐glia and macrophages/microglia, which jointly regulate injury repair and the recovery of neurological function. In the acute stage of injury, infiltrating macrophages and activated microglia secrete inflammatory factors such as TNF‐α and IL‐1, which can trigger hypertrophy reactions in NG2‐glia and upregulate the expression of NG2 proteoglycans [77]. In terms of spatial distribution, NG2‐glia and activated OX42^+^ macrophages/microglia are intermingled within the injured core region. Moreover, soluble factors secreted by macrophages/microglia (e.g., tissue inhibitor of metalloproteinase‐1 (TIMP‐1)) significantly suppress the proliferation and sphere formation of NG2‐glia in vitro, suggesting that the local inflammatory milieu may contribute to the long‐term failure of glial cell replacement in the lesion epicenter [78]. It is noteworthy that the NG2‐glia itself plays a pivotal role in the regulation of macrophage function. The absence of NG2‐glia reduces the infiltration of macrophages into the injury site and simultaneously impairs their repair function, resulting in myelin repair disorders [42]. Additionally, NG2‐glia and macrophages engage in bidirectional interaction: NG2‐glia support axon regeneration by expressing laminin and fibronectin—providing structural substrates—and help stabilize the axon tip during macrophage‐mediated degeneration [20]. Functionally, the absence of NG2‐glia exacerbates the activation of microglia/macrophages and leukocyte infiltration after TBI, indicating that NG2‐glia has a protective effect in restricting excessive inflammatory responses [79]. In the multiple sclerosis model EAE, NG2 is expressed not only in OPCs and pericytes of the CNS but also on immune cells such as peripheral macrophages. It influences the shift of T cells to T‐helper Type 2 (Th2)‐type responses by regulating the expression of IL‐12 in dendritic cells, thereby reducing the severity of the disease [80].

#### 3.3.3 Neurons

NG2‐glia establish direct synaptic connections with neurons under normal physiological conditions and receive synaptic inputs from glutamatergic and gamma‐aminobutyric acid (GABA)ergic neurons [34]. This distinctive structure empowers NG2‐glia to monitor the activity status of neurons in real time and promptly respond to changes in the neuronal microenvironment via multiple ion channels and neurotransmitter receptors expressed by themselves. The functional significance of these neuron‐NG2 synapses remains incompletely understood, but they may allow neurons to rapidly regulate NG2‐glia proliferation and differentiation in an activity‐dependent manner.

Despite the existence of synaptic connections under physiological conditions, evidence for spontaneous trans‐differentiation of NG2‐glia into neurons, particularly in the adult CNS, is extremely weak and highly controversial. It is essential to distinguish three fundamentally different scenarios: (1) Spontaneous differentiation: In the undisturbed adult CNS, lineage‐tracing studies consistently demonstrate that NG2‐glia produce virtually no neurons. Their default differentiation fate is the oligodendrocyte lineage [81, 82, 83]. (2) Injury‐induced plasticity: Following ischemic, traumatic, or neurodegenerative injury, some studies have reported that NG2‐glia can express neuronal markers (such as neuronal nuclei (NeuN), microtubule‐associated protein 2 (MAP2)) or adopt “neuron‐like” morphologies [47, 84, 85]. However, most of these observations lack rigorous lineage tracing (e.g., Cre‐loxP systems) and functional (electrophysiological) validation, making it impossible to rule out cell fusion or non‐specific marker expression. Current evidence indicates that injury signals alone are insufficient to drive efficient, stable trans‐differentiation of adult NG2‐glia into functionally integrated neurons. This so‐called “plasticity” more likely represents a stress response rather than a physiologically meaningful repair mechanism. (3) Forced reprogramming: In contrast to endogenous events, exogenous interventions yield markedly different outcomes. Viral vector‐mediated overexpression of transcription factors (such as SRY‐box transcription factor 2 [SOX2], NeuroD1, achaete‐scute family bHLH transcription factor 1 [Ascl1]) can directly convert reactive NG2‐glia (as well as astrocytes) into glutamatergic or GABAergic neurons in vivo [84, 86, 87, 88]. Pioneering work by Tai et al. [84, 89] demonstrated that in spinal cord injury models, SOX2‐reprogrammed NG2‐glia not only differentiate into neurons but also integrate into local circuits, significantly improving motor and bladder function. This opens exciting new avenues for in situ neural regeneration. However, this strategy faces safety and translational challenges, including the precision of viral delivery, tumorigenic risks associated with long‐term transgene expression, and whether reprogrammed cells can survive and maintain functionality over the long term. In summary, endogenous neuronal trans‐differentiation in the adult CNS is extremely limited, and exogenous transcription factor‐mediated forced reprogramming remains the only reliable approach to achieve functional neurogenesis at present. These two levels of plasticity are fundamentally distinct in both biological mechanisms and therapeutic applications.

## 4 Involvement of NG2‐Glia in CNS Injury

### 4.1 Operational Definition of Temporal Stages

Given substantial variability across injury paradigms, we define “early,” “middle,” and “late” stages based on dominant cellular behaviors rather than fixed time windows: early stage refers to the acute activation/proliferation phase (typically hours to ~3 days post‐injury, depending on model); middle stage refers to the migration, scar‐associated remodeling, and onset of lineage commitment phase (~1–2 weeks); late stage refers to chronic remodeling, remyelination, and stabilization (weeks to months). These boundaries differ substantially across injury types, as summarized in Table 1.

**TABLE 1:** Operational temporal boundaries and time‐dependent NG2‐glia responses across major CNS injury models.

Injury model	Early stage (pathological‐dominant)	Middle stage (transitional/remodeling)	Late stage (reparative‐dominant)	References
SCI	~0–3 days: rapid NG2‐glia activation, proliferation, and migration toward lesion border/core	~3–14 days: contribution to scar‐associated remodeling; CSPG‐rich matrix may inhibit axonal extension; marked spatial heterogeneity (core vs. border vs. spared white matter)	> 14 days to chronic: persistence in lesion niche; participation in partial remyelination/tissue remodeling. Under forced interventions (e.g., Sox2), reprogramming‐like outcomes reported; spontaneous neurogenic conversion mainly in restricted contexts (e.g., juvenile settings)	[85, 89, 90, 91]
TBI	~0–24 h: reactive hypertrophy (soma enlargement/process extension), rapid response near lesion margin; magnitude correlates with injury severity	~1–7 days: proliferative peak and lesion accumulation; participates with astrocytes in lesion containment/scar remodeling; pathway changes (e.g., β‐catenin) reported in subsets models	~7–30 days (or longer): density trends back toward baseline in some models; subset differentiates into oligodendrocytes and supports remyelination; long‐term scar‐associated persistence observed. Neuron‐like conversion generally requires exogenous induction	[92, 93, 94]
Ischemic stroke	~0–24 h: peri‐infarct hypertrophy/process remodeling; transient GFAP^+^/NG2^+^ populations	~1–14 days: increased proliferation (often peaking around week 1–2); transient astrocyte‐like NG2‐glia subpopulations in scar/peri‐infarct regions	~2–8 weeks: peri‐infarct repopulation and oligodendroglial differentiation supporting remyelination; exosome‐associated pro‐repair signaling reported. NeuN/NG2 co‐label observations exist but require strict lineage validation	[13, 16, 47, 95]
Demyelination (EAE/cuprizone)	~0–3 days (lesion phase): robust NG2‐glia proliferation; GPR17 upregulation as differentiation checkpoint; early lesion recruitment	~3–10 days: migration into lesions and lineage progression begins; inflammatory context (especially EAE) can restrain maturation and produce regional heterogeneity	~10–21 days and beyond: new oligodendrocyte generation and remyelination (robust in cuprizone recovery, often incomplete in EAE); residual NG2‐glia pool remains for future repair	[47, 96, 97]

### 4.2 Early Stage: Activation and Pathological Involvement of NG2‐Glia

#### 4.2.1 Dynamic Changes in Proliferation and Migration

After CNS injury, NG2‐glia proliferation and migration exhibit highly dynamic spatiotemporal characteristics. In the early phase of ischemic injury, NG2‐glia are rapidly activated and proliferate around the infarction core, briefly forming a unique “astrocyte‐like” subpopulation within the glial scar [16]. This proliferative response varies across brain regions. For example, 7–14 days after ischemia, NG2‐glia in the hippocampal CA1 pyramidal cell layer display a contracted morphology, whereas those in the adjacent radiation layer show complex branching [17]. Regarding migration dynamics, in vivo two–photon imaging studies have revealed that NG2‐glia exhibit slow yet continuous migration after electrode implantation or puncture: 12 h after injury, cell processes begin to extend toward the lesion, followed by directional cell body migration at approximately 1.6 μm/h. The activation zone expands to about 190 μm from the injury interface within 72 h [15].

#### 4.2.2 CSPG Secretion and Axonal Regeneration Inhibition

In the early stage of CNS injury, NG2‐glia are promptly activated and undergo substantial proliferation, and the expression of NG2/CSPG4 is notably upregulated [98]. Importantly, reactive astrocytes have also been verified to produce NG2/CSPG4, with significantly increased secretion levels post‐injury, suggesting diverse cellular sources of CSPG within glial scars [98]. At the molecular regulatory level, the transcription factor old astrocyte specifically induced substance (OASIS) is induced to be expressed in proximal reactive astrocytes after injury and directly participates in regulating the gene expression of multiple CSPG core proteins, including NG2, thereby establishing a microenvironment that is unfavorable for axon growth. Beyond the traditional “chemical barrier” concept, recent studies have proposed additional mechanisms: NG2‐glia can form synaptic‐like connections with dystrophic axon terminals, arresting axon regeneration through “physical entrapment”. The application of Chondroitinase ABC can effectively eliminate the inhibitory effect of CSPG and relieve this binding [21]. More recent work has focused on CSPG‐mediated integrin signaling and intracellular RhoA/ROCK activation as downstream mechanisms of regeneration failure [99]. Additionally, damage‐related molecules such as myelin fragments further stimulate NG2/CSPG4 expression in infiltrating macrophages, amplifying the early inhibitory effect [98].

#### 4.2.3 The Role in Neuropathic Pain

Beyond establishing a physical barrier for axonal regeneration via CSPG secretion, NG2‐glia may directly participate in central sensitization of pain circuits through the secretion of inflammatory mediators. Our recent study demonstrated that following peripheral nerve injury (PNI), NG2‐glia in the spinal dorsal horn significantly upregulate IL‐24. Concurrently, TNF‐α induces NG2‐glia to upregulate the IL‐24 receptor IL‐20R2. Intrathecal injection of neutralizing antibodies or siRNAs targeting IL‐24 or IL‐20R2 effectively alleviates mechanical and thermal hyperalgesia in mice [100]. Additionally, NG2‐glia establish direct synaptic connections with neurons, exerting regulatory control over neuronal excitability and influencing synaptic transmission [34]. Considering that alterations in neuronal synaptic plasticity serve as a fundamental pathological basis for chronic pain, including neuropathic pain [101], and acknowledging the involvement of NG2‐glia in inflammatory responses, we hypothesize that NG2‐glia may play a pivotal role in modulating neuropathic pain. Currently, there are scarcely any studies on the role of spinal NG2‐glia in neuropathic pain. Hence, in‐depth research on the function and mechanism of spinal NG2‐glia in neuropathic pain not only has significant innovative value but may also lead to new breakthroughs in the understanding of this field.

### 4.3 Middle and Late Stages: Multi‐Dimensional Exploration of the Repair Potential of NG2‐Glia

#### 4.3.1 Myelin Regeneration Capacity

NG2‐glia' differentiation into mature oligodendrocytes is the core link of myelin regeneration after injury. However, the efficiency of endogenous myelin regeneration is limited. The majority of NG2‐glia face challenges in completing terminal differentiation and often remain in a precursor state. Recent studies have found that this process is precisely regulated by adjacent glial cells. Sherafat et al. [102] discovered that neurociliin‐1 (Nrp1) expressed by microglia can promote the expansion of NG2‐glia and subsequent myelin repair after demyelinating injury by transactivating the PDGFRα receptor on the surface of NG2‐glia. This finding emphasizes that myelin regeneration is not solely an autonomous process of NG2‐glia but rather depends on cooperative interactions within the glial network. Sánchez de la Torre et al. used a conditional knockout mouse model to show that CB1 receptors on NG2‐glia are essential for myelin regeneration. By inhibiting the RhoA/ROCK/Cofilin pathway via Gi/o proteins, CB1 promotes NG2‐glia differentiation into oligodendrocytes and enhances myelination [33]. Despite these promising targets, endogenous remyelination faces fundamental limitations. The newly formed myelin sheaths are typically thinner, less dense, and have shorter internodes than original myelin. More critically, a recent study by Mironova et al. redefines the limitations of endogenous myelin repair by revealing that OPCs do not increase their differentiation rate after injury. Instead, they undergo “constitutive differentiation” at a fixed, injury‐insensitive frequency—even during active demyelination [103].

#### 4.3.2 Neural Trans‐Differentiation Potential

The traditional view posits that NG2‐glia predominantly differentiate into glial lineage cells. However, recent studies have provided evidence for their capacity to trans‐differentiate into neurons, although this process in the adult CNS remains inefficient and controversial. Zhao et al. [85] found that NG2‐glia can form oligomeric spheres and trans‐differentiate into neuron‐like cells post‐SCI, with concurrent upregulation of keratin expression. More importantly, Tai et al. [84] employed SOX2 overexpression technology to achieve in vivo reprogramming of NG2‐glia in adult mice, enabling differentiation into excitatory and inhibitory spinal intrinsic neurons that establish synaptic connections with ascending and descending spinal pathways. However, it is important to note that these trans‐differentiation phenomena are predominantly observed under conditions of exogenous transcription factor overexpression or in juvenile animals; under normal physiological or injury‐only conditions in adults, such events are rare.

Adding to this complexity, different injury types exert differential regulatory effects on NG2‐glia differentiation fate. Kirdajova et al. [16], using single‐cell transcriptome analysis, found that a transient “astrocyte‐like” NG2 subpopulation expressing glial fibrillary acidic protein (GFAP) emerges only after permanent cerebral ischemia, whereas in models of stab injury and demyelination, the oligodendrocyte maturation lineage predominates. Furthermore, a systematic review by Rigo et al. [92] concluded that through various pharmacological and genetic manipulations, NG2‐glia can be induced to differentiate into neurons in models of brain trauma and SCI, leading to improved functional outcomes. Collectively, while the neurogenic potential of NG2‐glia in the adult CNS is in a latent state, it can be activated via exogenous intervention, opening new avenues for in situ neural regeneration.

#### 4.3.3 Anti‐Stress and Protective Effects

A review systematically summarized the diverse functions of NG2‐glia beyond myelin formation, including regulation of the metabolic environment, direct interaction with neurons, maintenance of blood–brain barrier integrity, and modulation of inflammatory responses [104]. In the context of neurodegenerative diseases associated with neuroinflammation, NG2‐glia assume both protective and pathological roles [18, 105, 106]. Specifically, they can regulate microglial activity and facilitate the transformation of inflammatory phenotypes into anti‐inflammatory ones [18]. However, their abnormal activation can also exacerbate scar formation and neuronal degeneration [15]. Recent work has also begun to explore the role of NG2‐glia in oxidative stress responses and mitochondrial transfer to neurons [107], although these findings remain preliminary and require further validation.

### 4.4 Pro‐Repair and Restrictive Roles of NG2‐Glia Across Early, Middle, and Late Phases

To integrate the stage‐specific evidence discussed above, we summarize the pro‐repair and pathological/restrictive roles of NG2‐glia across early, middle, and late phases in Table 2. This framework reveals several important patterns. First, the early‐stage “containment versus inhibition” paradox suggests that therapeutic interventions aimed at eliminating CSPG‐mediated barriers must be carefully timed to avoid disrupting beneficial wound compaction. Second, the middle stage emerges as the most context‐dependent phase, where outcomes are heavily shaped by regional heterogeneity and immune tone—implying that successful therapies may need to be tailored to specific injury types and microenvironments. Third, the late‐stage evidence strongly supports remyelination and tissue remodeling, while endogenous neurogenesis remains poorly supported and generally requires exogenous manipulation (e.g., SOX2 reprogramming). Collectively, this stage‐based analysis underscores that NG2‐glia should be viewed not as intrinsically “good” or “bad” cells, but as dynamic regulators whose net contribution depends critically on injury context, regional heterogeneity, and temporal dynamics.

**TABLE 2:** Stage‐dependent controversial roles of NG2‐glia in CNS injury.

Stage	Pro‐repair evidence	Pathological or restrictive evidence	Key caveat	Key references
Early stage	Rapid accumulation of NG2‐glia may contribute to wound compaction/lesion containment; early interaction with microglia/macrophages may help organize the local response	CSPG‐rich extracellular matrix can form a chemical and physical barrier to axonal regrowth; early inflammatory cues can drive reactive changes that are not regeneration‐permissive	Early containment and early growth inhibition can occur simultaneously, so the net effect depends on injury context and readout	[98, 108]
Middle stage	NG2‐glia migrate toward the lesion border, participate in boundary organization, and begin oligodendroglial lineage progression; this stage is a transition toward repair in permissive environments	Persistent lesion‐core inflammation may suppress proliferation, sphere formation, or maturation; scar‐associated NG2‐glia may remain part of a non‐permissive matrix	This is the most context‐dependent phase, with outcome shaped by regional heterogeneity and immune tone	[76, 108, 109]
Late stage	NG2‐glia support remyelination and long‐term tissue remodeling; under forced reprogramming (e.g., SOX2), can be induced to generate functional neurons, improving functional recovery	Broad spontaneous neurogenic conversion in the adult CNS remains weakly supported; remyelination is often incomplete in chronic inflammatory lesions; intrinsic lineage barriers such as Olig2 can limit fate switching	The strongest evidence supports remyelination and remodeling, whereas endogenous neurogenesis remains context‐restricted and often manipulation‐dependent	[47, 108, 109, 110]

## 5 Therapeutic Applications of NG2‐Glia

In recent years, NG2‐glia, being an important target for the repair of CNS injuries, has witnessed significant progress in their therapeutic strategies. We have summarized these strategies, as presented in Table 3.

**TABLE 3:** Therapeutic strategies targeting NG2‐glia for CNS injury repair.

Strategy	Target/tool	Mechanism of action	Therapeutic effect	Limitations/challenges	Temporal note	References
Promoting remyelination	CB1 receptor agonists (e.g., WIN55212‐2)	Inhibits RhoA/ROCK/Cofilin pathway via Gi/o protein; promotes NG2‐glia differentiation into mature oligodendrocytes	Enhances myelination, protects axons, improves neurological function	Potential psychiatric side effects of systemic CB1 activation; requires local delivery	Middle‐to‐late‐stage promotion may be preferable	[33]
	Microglial Nrp1	Trans‐activates PDGFRα receptor on NG2‐glia	Promotes NG2‐glia expansion and subsequent myelin repair	Nrp1 is widely expressed in other cell types; poor targeting specificity	Middle‐to‐late‐stage promotion may be preferable	[102]
	PDGF/PDGFRα agonists	Directly stimulates NG2‐glia proliferation; increases NG2‐glia pool	Increases NG2‐glia available for myelin repair	May exacerbate glial scar formation; requires precise dosing and timing	Early‐stage inhibition may be preferable	[111]
	Endocannabinoid modulators	Promotes NG2‐glia differentiation and myelination via CB1 receptors	Harnesses endogenous system; fewer exogenous side effects	Complex mechanisms involving multiple cannabinoid receptors	Middle‐to‐late‐stage promotion may be preferable	[33]
Neutralizing inhibitory microenvironment	Chondroitinase ABC (ChABC)	Degrades CSPG glycosaminoglycan chains; relieves chemical barrier	Reduces CSPG‐mediated chemical inhibition and physical entrapment of axons	Poor enzyme stability; short half‐life; delivery difficulties; no effect on OPC “constitutive differentiation” frequency	Early‐stage inhibition may be preferable	[21]
	CSPG receptor blockers (e.g., protein tyrosine phosphatase sigma (PTPσ) inhibitors)	Blocks binding of CSPGs to neuronal receptors (PTPσ, leukocyte common antigen‐related receptor (LAR), etc.)	Reduces axonal growth inhibition; promotes regeneration	Requires identification of injury‐specific CSPG variants; may affect normal CSPG functions	Early‐stage inhibition may be preferable	[112]
Reprogramming into neurons	SOX2 overexpression	Trans‐differentiates NG2‐glia into excitatory and inhibitory spinal intrinsic neurons	Generates functional synaptic connections; reduces glial scar; improves motor and bladder function	Safety concerns (tumorigenesis, off‐target differentiation); requires optimized viral vectors and delivery	Not done/not mentioned	[84, 86]
	NeuroD1 overexpression	Reprograms NG2‐glia (and reactive astrocytes) into glutamatergic and GABAergic neurons	Achieves functional integration into neural circuits	Same concerns as SOX2; requires validation of specificity in NG2‐glia	Not done/not mentioned	[87, 88]
	EGFR inhibitors (e.g., Gefitinib)	Inhibits EGFR signaling pathway; induces neuronal phenotype	Promotes NG2‐glia‐to‐neuron differentiation; improves behavioral outcomes	Mechanism not fully understood; may affect other EGFR‐dependent cellular functions	Not done/not mentioned	[113]
	SOX6 inhibition	SOX6 maintains NG2‐glia quiescence by suppressing Neurog2 expression; relieving this inhibition promotes neurogenesis	Unlocks endogenous neurogenic potential	SOX6 has important functions in other cell types; high risk of systemic inhibition	Not done/not mentioned	[114]
Anti‐inflammatory/neuroprotection	TGF‐β2/TGFBR2 axis activation	NG2‐glia‐derived TGF‐β2 inhibits microglial overactivation via microglial TGFBR2	Reduces neuroinflammation; protects neurons; maintains immune homeostasis	May also suppress beneficial immune responses; high risk of systemic application	Early‐stage inhibition may be preferable	[18]
	HGF/Met signaling enhancement	NG2‐glia‐derived HGF inhibits microglial IL‐1β expression	Protects neurons; suppresses neuroinflammation	Short half‐life of HGF; poor blood–brain barrier penetration	Early‐stage inhibition may be preferable	[115]
	Act1/IL‐17 pathway blockade	Act1 mediates IL‐17‐induced inflammatory gene expression in NG2‐glia	Reduces NG2‐glia‐mediated neuroinflammation	Validated in EAE model; requires further validation in other injury models	Early‐stage inhibition may be preferable	[115]
Combinatorial strategies	CB1 agonist + ChABC	Simultaneously promotes OPC differentiation and relieves CSPG‐mediated inhibition	Synergistically enhances remyelination and functional recovery	Complex dual delivery; long‐term safety unknown	Middle‐to‐late‐stage promotion may be preferable	Preclinical stage
	SOX2 + anti‐CSPG antibodies	Promotes neuronal trans‐differentiation and relieves axonal growth inhibition	Enhances efficiency of neuronal generation and functional integration	Requires validation of synergistic effects; high delivery difficulty	Not done/not mentioned	Further studies needed
	PDGF + TGF‐β2	Promotes NG2‐glia proliferation while modulating inflammatory microenvironment	Increases repair cells while controlling inflammation	Potential temporal conflict between the two actions; requires precise regulation	Early‐stage inhibition may be preferable	Further studies needed

### 5.1 Promotion of Remyelination

Given that NG2‐glia possess the potential to spontaneously recruit and differentiate into mature oligodendrocytes after CNS injury, they have emerged as a highly promising intervention target for regenerative therapy in demyelinating diseases. For example, in the mouse SCI model, the OPC induced by WIN55212‐2 underwent robust differentiation into functional oligodendrocytes and formed myelin sheaths both in vitro and in vivo [116]. Selective CB1 receptor knockout in NG2‐glia impaired oligodendrocyte regeneration and functional myelin repair, and exacerbated axonal injury in a toxin‐induced demyelination model [33]. In multiple sclerosis, endogenous NG2‐glia differentiate into oligodendrocytes, which remyelinate partially exposed axons [117]. OPCs modulate immune response and early demyelination in a murine model of EAE [118].

Despite these promising findings, several fundamental limitations must be acknowledged. The newly formed myelin sheath is typically thinner, less dense, and has shorter internodes—reflecting the functional limits of endogenous repair. More critically, a recent study demonstrated that OPCs do not increase their differentiation rate after injury; instead, they undergo “constitutive differentiation” at a fixed, injury‐insensitive frequency [119]. This discovery fundamentally redefines the bottleneck of endogenous remyelination: it is not merely that the microenvironment is inhibitory, but that OPCs themselves lack an injury‐responsive differentiation program.

### 5.2 Enhancement of Neuronal Regeneration

A recent study demonstrated that in clinically relevant spinal cord contusion models, reprogramming NG2‐glia with SOX2 transcription factors not only induces the generation of new neurons and reduces glial and fibrotic scar formation but also significantly ameliorates neurogenic bladder dysfunction [84]. Additionally, NG2‐glia promote neural regeneration in young mice with SCI by spontaneously transdifferentiating into neuron‐like cells and upregulating keratin expression [85]. Further in‐depth exploration shows that SOX6 preserves the quiescent state of the NG2‐glia subset by suppressing the expression of the neurogenic gene Neurog2 [114]. Meanwhile, findings from astrocyte reprogramming studies indicate that inducible barriers analogous to Olig2 are activated during cell fate transitions to oppose reprogramming [120]. This implies that when employing NG2‐glia for regenerative therapy, combined intervention targeting these inhibitory molecules (such as SOX6 or Olig2) may further improve the efficiency of neuronal conversion and functional integration. However, safety concerns remain, including the risk of tumorigenesis from uncontrolled proliferation, off‐target differentiation, and the potential for glial scar disruption that could compromise lesion containment. Rigorous preclinical safety studies are therefore essential before clinical translation. Critically, it must be emphasized that the therapeutic potential for neuronal regeneration currently relies almost exclusively on forced reprogramming strategies, as endogenous neurogenesis from NG2‐glia in the adult CNS is insufficient to support meaningful functional recovery.

## 6 Future Studies

Despite substantial progress in understanding NG2‐glia biology, several critical questions remain unresolved and warrant dedicated investigation. First, what drives the heterogeneity of NG2‐glia across CNS regions and injury types—is it determined by distinct developmental origins or by local microenvironmental cues? Single‐cell and spatial transcriptomic profiling across different injury paradigms and time points will be essential to map functional subpopulations. Second, what are the upstream signals that trigger the rapid proliferation and migration of NG2‐glia immediately after injury? While Notch signaling and blood–brain barrier disruption have been implicated, the initiating molecular events remain unknown. Third, why does the adult CNS environment convert NG2‐glia from efficient remyelinating progenitors into largely quiescent bystanders? Identifying the missing developmental or injury‐induced signals could unlock their endogenous repair capacity. Fourth, can neurons newly generated from NG2‐glia (either spontaneously in juvenile animals or via forced reprogramming in adults) functionally integrate into existing neural circuits? Advanced lineage tracing, optogenetics, and long‐term in vivo imaging are required to address this question. Fifth, how can the “constitutive differentiation” limitation of NG2‐glia—their strong predisposition toward oligodendrocyte lineage at the expense of other fates—be overcome for therapeutic remyelination? Directly manipulating intrinsic transcriptional programs, such as by targeting the Olig2 barrier identified in recent reprogramming studies, may offer a promising strategy. Addressing these questions will require interdisciplinary approaches integrating single‐cell technologies, genetic lineage tracing, gene editing, biomaterials, and functional imaging, while remaining grounded in fundamental developmental and injury biology.

## 7 Conclusion

NG2‐glia are emerging as dynamic, context‐dependent regulators of CNS injury and repair, rather than simply beneficial or detrimental players. The time‐dependent dual‐role framework proposed in this review—distinguishing early‐stage pathological involvement (e.g., CSPG secretion, neuropathic pain facilitation) from middle‐to‐late‐stage reparative potential (e.g., remyelination, neuroprotection, and inducible neurogenesis)—provides a useful conceptual foundation for understanding the complex and often contradictory functions of these cells. Mechanistically, age, microenvironmental signals, and intrinsic transcriptional programs jointly determine the fate and functional state of NG2‐glia across different injury stages and models. Therapeutically, our analysis suggests that successful strategies will likely require precise, stage‐specific targeting rather than unregulated promotion or inhibition of NG2‐glia activity, and that interventions must account for the substantial differences in NG2‐glia behavior across injury types (e.g., TBI vs. SCI vs. ischemic stroke). Future investigations integrating emerging technologies—including single‐cell multi‐omics, advanced lineage tracing, and in vivo functional imaging—will be essential to translate this conceptual framework into effective and safe regenerative therapies. Ultimately, a deeper understanding of the molecular logic governing NG2‐glia state transitions will inform the next generation of precision medicine approaches for CNS injuries.

## Author Contributions

G.C. conceptualized the topics. G.C. and Y.C. wrote and revised the manuscript. X.S., M.L., and J.L. searched for literature. All authors made significant contributions to the manuscript's revision, and the final version was unanimously approved.

## Funding

This study was supported by the National Natural Science Foundation of China (32271054, 32571189).

## Conflicts of Interest

The authors declare no conflicts of interest.

## Data Availability

Data sharing not applicable to this article as no datasets were generated or analysed during the current study.
